# The global tilt: a new pillar in the radiological assessment of vertebral fracture risk

**DOI:** 10.1590/0100-3984.2025.58.e6-en

**Published:** 2025-11-07

**Authors:** André Yui Aihara

**Affiliations:** 1 Professor Adjunto do Departamento de Diagnóstico por Imagem da Escola Paulista de Medicina da Universidade Federal de São Paulo (EPM-Unifesp), São Paulo, SP, Brazil.

In the dynamic field of clinical radiology, our mission goes beyond simply identifying abnormal findings. The mission is to provide diagnostic information that informs clinical decisions and, ultimately, improves the quality of life of our patients.

Osteoporosis, an insidious disease affecting millions—predominantly postmenopausal women—exemplifies our responsibility as clinical radiologists. Vertebral fragility fractures, devastating complications of osteoporosis, not only cause severe pain and deformities but also constitute a robust predictor of morbidity and mortality. Worldwide, one in three women and one in five men over the age of 50 will experience osteoporotic fractures in their lifetime^(1)^.

Diagnostic imaging, with its unique ability to visualize the skeleton and its complex alterations, remains the cornerstone in assessing the risk of and detecting such fractures^(2^,^3)^.

Although bone densitometry is traditionally the primary tool for quantifying bone mineral density (BMD) and bone loss, the intrinsic biomechanical complexity of the spine and the multifactorial nature of fragility fractures call for a more holistic approach. It is in this context that the analysis of sagittal spinopelvic alignment, accessible through radiographs, emerges as an indispensable component, enriching the assessment of BMD with a functional and structural perspective^(3^,^4)^.

In this context of continuous diagnostic advancement, the article “Correlation between spinopelvic sagittal balance and vertebral fractures in postmenopausal women”^(5)^, published in our well-respected journal, **Radiologia Brasileira**, emerges as a valuable contribution. The study, carefully conducted and involving 93 postmenopausal women with osteopenia or osteoporosis, details the analysis of spinopelvic parameters obtained through panoramic radiographs of the spine and pelvis. The central, relevant finding is the statistically significant correlation between the global tilt (GT) and the presence of vertebral fractures. The finding that “for each 1-degree increase in GT, the prevalence of fracture increased, on average, by 2.1%” indicates that the GT is an independent, clinically useful prognostic indicator. By integrating pelvic retroversion and trunk anteversion, the GT offers a robust, comprehensive measure of global alignment. However, it is essential to recognize the methodological limitations of the study, such as its cross-sectional design, which prevents direct inferences of causality, and the limited focus on a specific population (women with osteopenia/osteoporosis, without assessment of lower limb compensation or the inclusion of individuals with normal BMD).

The results presented by the authors of the study of interest^(5)^ pave the way for promising developments, in the scientific literature and in radiological practice. The identification of GT as a predictor of vertebral fractures suggests that its measurement could be incorporated into the routine for the evaluation of patients with osteoporosis or osteopenia, complementing the diagnostic arsenal that includes bone densitometry and the identification of clinical risk factors other than low BMD. It is imperative that radiologists become familiar with the technique for measuring GT and learn how to interpret the result. In addition, the study highlights the need for longitudinal research to follow patients over time, which could firmly establish the cause-and-effect relationship between GT changes and the incidence of new fractures. Given the global impact of osteoporosis, future studies including individuals with normal BMD, as well as investigating the applicability of these findings to male patients and other populations, are essential to expanding our understanding of the condition. From a technological perspective, the continued evolution of systems such as the EOS imaging system—which offers full-body scans with low radiation doses and the ability to visualize compensatory mechanisms—promises an era of diagnoses that are even more detailed and personalized. By integrating this new understanding of sagittal balance into our practice, we not only raise our diagnostic standards but also strengthen the fundamental role of radiology in the prevention and effective management of fragility fractures, contributing to making medical practice truly more predictive and preventive.
